# Enabling high-resolution wildlife tracking: antenna beam-based approach including novel per-position error estimations for automated radiotelemetry systems

**DOI:** 10.1186/s40462-026-00687-1

**Published:** 2026-08-05

**Authors:** Mirjam R. Rieger, Jan Schmitt, Paula Machin, Johann Musculus, Ralf Dittrich, Thomas K. Gottschalk, Jannis Gottwald, Patrick Lampe, Jonas Höchst

**Affiliations:** 1 University of Applied Forest Sciences Rottenburg, Schadenweilerhof, Rottenburg am Neckar, 72108 Germany; 2 Eberhard Karls University Tübingen, Auf der Morgenstelle 28, Tübingen, 72076 Germany; 3 Eurofins MITOX BV, Science Park 408, Amsterdam, 1098XH Netherlands; 4 trackIT Systems GmbH, Unterm Bornrain 4, Cölbe, 35039 Germany

**Keywords:** Automated Radiotelemetry System, Position finding, Position error, VHF, Radiotracking, Wildlife movement

## Abstract

**Background:**

The increasing importance of wildlife movement data in ecology and conservation has fueled the development of Automated Radiotelemetry Systems (ARTS) using very-high-frequency (VHF) transmitters. To make optimal use of this data, highly precise analysis methods are needed to detect even small-scale movement changes and thus provide high data quality. While various approaches have successfully minimized position errors in ARTS, they mostly rely on a single mean error estimate.

**Methods:**

We present two contributions. First, an antenna geometry-based position finding method (antenna beams) for small scale movements that reduces position errors (PE) and increases the number of position estimates. Second, a model for per-position error estimation, predicting error as a function of signal and position characteristics, applicable for data without ground-truth information and across various position finding methods. Using ground-truth data from VHF transmitters recorded simultaneously with the ARTS trackIT and GPS, we validated and compared yield, position errors and predictive performance of our approach with the common angulation and multilateration methods.

**Results:**

Our antenna beam-based method provided a substantial alternative to angulation for directional set-ups, achieving comparable mean PEs (41 m vs. 44 m) and especially higher number of localizations (up to 99% vs. 30 to 66%). The per-position error estimation model demonstrated a strong predictive performance (mean absolute deviation from true error down to 21 m) utilizing parameters such as the number of participating stations and antennas, maximum signal strength, normalized summed up signal strengths and positioning within the study area.

**Conclusions:**

Our results indicate that (i) the antenna beam-based position-finding method outperforms common methods in both accuracy and yield, (ii) the novel introduced per-position error estimation model reliably reflects measured PE from ground-truth data, and (iii) the resulting setup provides a robust foundation for high-resolution wildlife movement analyses.

**Supplementary information:**

The online version contains supplementary material available at 10.1186/s40462-026-00687-1.

## Background

The recognition of movement patterns of wild animals is becoming an increasingly important component in better understanding population dynamics and as a basis for decision-making in nature conservation and landscape management [1]. This high demand for movement data in wildlife conservation led to the development of a variety of automated telemetry systems [2–5] and ecologists face an unprecedented wealth of data, also termed the ‘golden age of animal tracking’ [1]. Ensuring data quality and position accuracy across emerging systems is challenging due to differences in hardware, software, and data formats. Thus, in parallel with developing telemetry system hardware, the analytical methods for movement data must also be optimized to enable the validation, precision, handling, and processing of large amounts of data.

The two most common technologies for recording wildlife movement data are (i) the Global Positioning System (GPS) and (ii) very high-frequency (VHF) telemetry [6]. Widely used **GPS systems** use receivers that measure the time of arrival of incoming satellite signals (so called time-based GPS). Satellites make GPS immediately operational across large areas of the world, providing reliable positioning with highly synchronized clocks [5]. However, GPS receivers rely on heavy hardware components for data collection and storage (transmitter weights usually start at 6 g, recent developments using low range communication start at 1.5 g [7]). They often interfere with the rule of transmitters not exceeding five percent of the animal’s body weight to avoid impact on natural behavior, prohibiting their use for about 60% of vertebrates [8]. Therefore, Automated Radiotelemetry Systems (ARTS) using 2.4 GHz or **VHF technology** with lightweight transmitters of less than 0.1 g have extended the scope of radiotelemetry systems to many small animals, like songbirds, bats, or insects [2–4, 9–11]. ARTS rely on a network of passive ground stations with receivers distributed throughout the study area, allowing us to continuously track multiple animals at once and providing high flexibility for different-sized areas. Stations are either equipped with a single omnidirectional antenna, which uniformly receives signals from all directions within a 360-degree radius, or multiple directional antennas, each primarily receiving signals from their respective orientation. The most comprehensive ARTS, the Motus Wildlife Tracking System, operates a collaborative network of more than 300 receiving stations on three continents [2] and documents large-scale movements such as bird and bat migration (Motus; https://motus.org). At the regional and landscape level, ARTS operate with fewer receiving stations, aiming to monitor small-scale movements of animals, which requires a more accurate positioning than the global Motus system [12], with design and structure (e.g. which and how many stations to use) tailored to the study question. Once users overcome the hurdles of individual configuration (e.g., factors impairing radio signal transmission such as dense vegetation cover and moist climate [4]), such ARTS can provide a large amount of movement data.

Once the raw radio detection data has been collected, there still remains the critical goal of producing high-resolution movement patterns. One major reason why ARTS are less accurate than time-based GPS is that positions are mainly calculated based on physical correlation of sender-receiver distance and received signal strength (RSS) which is prone to imprecision. Such imprecision can arise from several sources, e.g., the underlying hardware and spatial distribution of the autonomous receiving stations, signal strength of used transmitters, topography and vertical landscape elements of the study area, the behavior of the animal itself (ground-dwelling, flying, underground), and man-made signal noise from nearby electronic sources [1, 4, 13]. Common position finding methods involve (i) (tri)angulation using directional stations (e.g. [3, 9]) (ii) (multi)lateration using directional or omnidirectional stations (e.g. [14, 15]), (iii) RSS fingerprinting and grid search methods using directional or omnidirectional stations [14, 16, 17], or (iv) time of arrival (similar to GPS) using omnidirectional stations [18, 19]. Depending on the setup and method used, the mean position error derived from ARTS studies therefore covers a wide spectrum, ranging from around 5 m (lateration by [15], time of arrival by [18]), 15 m (angulation by [9]), 30 m (RSS fingerprinting by [16]), 43 m (lateration by [16]), 50 m (multilateration by [20]), or 105 m (trilateration by [21]), over 300 m (RSS fingerprinting by [14]) or 500 m (angulation by [14]) up to 1 to 15 km for large-scale ARTS ([2], antenna beams by [22], see Supplement 2.4 for an overview). Some methods, especially angulation, additionally have high requirements for signal detection, leading to data loss when these requirements are not met [14]. Filters aiming at reducing mean position errors additionally exclude positions prone to high errors, e.g., with low signal strengths, further limiting usable data [20]. Additionally, methods testing positioning usually result in only one mean position error for the whole system, but the individual per-position error can vary greatly, especially increasing as the distance between a transmitter and the receiving station increases [14, 16]. Unlike GPS, ARTS are thus not a ‘one-fits-all’ solution, but every set-up has to be customized to the study requirements needed regarding the quality and quantity of position estimates, ideally modeling the position error based on calibration data [23]. Therefore, conclusions about wildlife movements might be biased if users do not sufficiently test their given set-up or simply assume that their data are error-free, especially, if the position error exceeds the studied movement patterns [23].

The aim of our study is therefore to improve position estimation, reduce position errors, and offer per-position error estimations of ARTS data, thereby generating high-precision movement data with a temporal resolution of seconds and a spatial resolution on the scale of tens of meters. Using ground-truth data from VHF-transmitters that were simultaneously recorded with a GPS device and an ARTS, we optimize and compare estimated positions and their position errors between the two common position finding methods angulation and multilateration with an approach described in [12]. This approach (hereafter referred to as antenna beams) estimates positions along the lobe of the receiving antenna and averages multiple positions based on receiving signal strength and is tested for directional and omnidirectional stations. In a final step, we model position error as a function of different signal and position characteristics such as number of participating stations and antennas as well as maximum signal strength, normalized summed up signal strengths and positioning within the study site, to predict errors for position estimates without ground-truth data, i.e., data from the animal studied. These predicted per-position errors can then be used for further data analysis, such as home ranges or habitat use. We recorded and analyzed data using the trackIT ARTS by [13], but the accompanying code and formulae of our work ensure that the workflow can be adapted to telemetry data recorded with other ARTS.

## Material and methods

### Study area

**Fig. 1:**
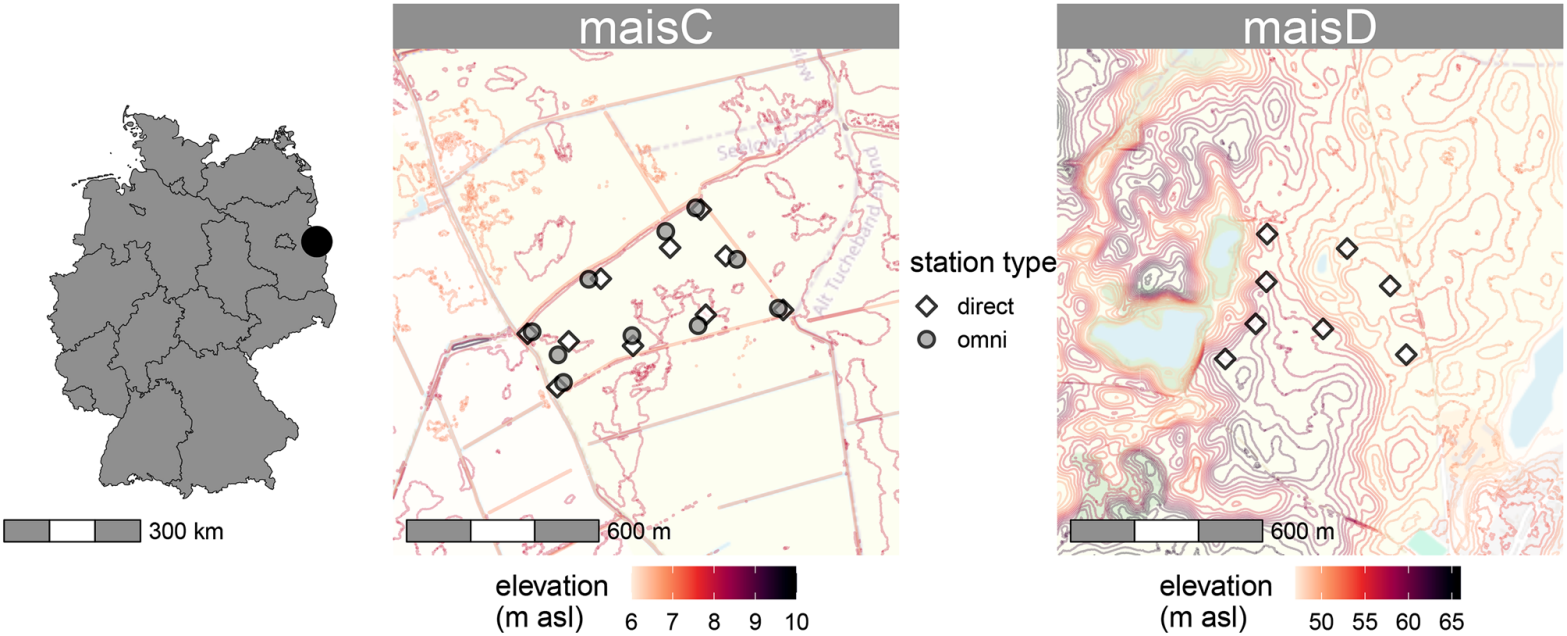
Study area (black point, left) with sites maisC (middle) and maisD (right) in Märkisch Oderland (Brandenburg, Germany) including the station set-up. Elevation is given in isolines in 1 m-steps. Copyright map data: OpenStreetMap contributors

The study was part of a project that investigated the use of maize fields by songbirds and was carried out in two agricultural areas 70 km east of Berlin in the Märkisch Oderland district in Brandenburg, Germany (Fig. 1, left) in late summer and autumn 2023. Both sites were dominated by agricultural land (maize, harvested grain, soy) and also contained woody structures such as tree lines, hedges and shrubs, as well as ditches or lakes with accompanying reed vegetation. Site *maisC* was located in the Oderbruch with only minor elevation differences, while the site *maisD* was located in Lubusz land, a region with moderate elevation differences with up to 15 m difference in altitude (Fig. 1, Supplement 1.2).

### Automated radiotelemetry

**Fig. 2:**
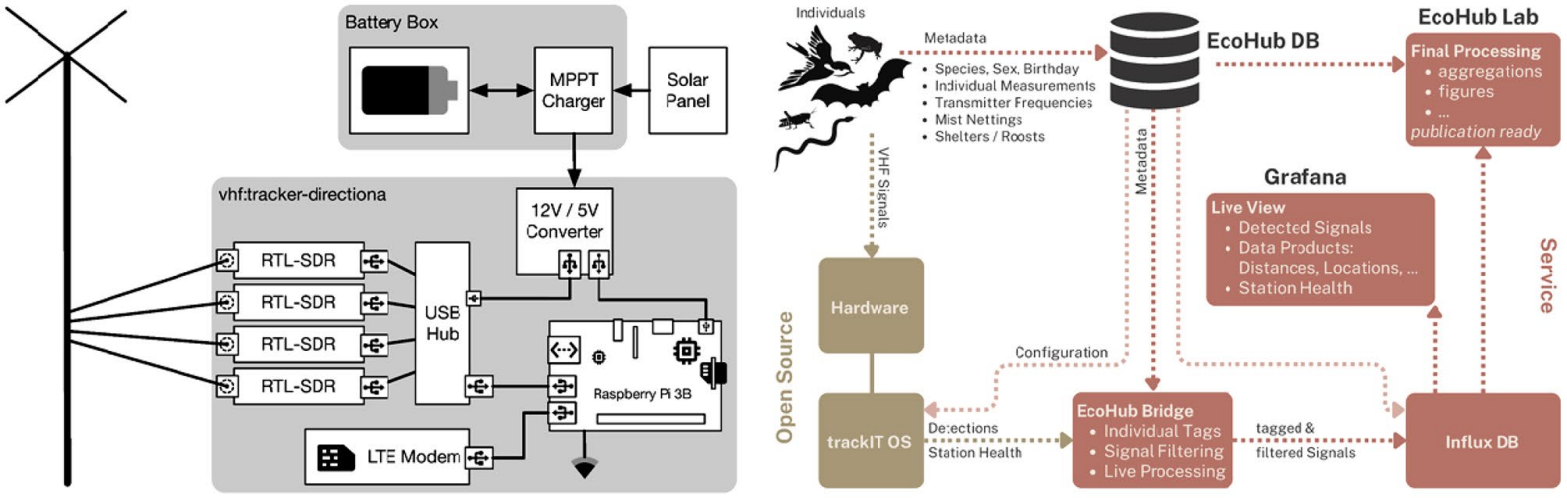
*Left*: Commodity off-the-shelf hardware components of a directional VHF station. *Right*: The system architecture and components of the trackIT ARTS

At both sites, we set up a network of automatic radiotelemetry stations (Fig. 1). For maisC we used a combined set-up of ten directional stations (each with four directional antennas) and ten omnidirectional stations (each with one omnidirectional antenna), with the area enclosed by the stations totaling 20 ha, and for maisD we used a set-up of eight directional stations, covering a core area of 16 ha. Figure 2 left shows the hardware components used for the directional stations.

The stations are operated with trackIT OS version 2023.05.3 (trackIT Systems, Cölbe, Germany), which is available under an open source license^1^. The stations were configured to detect VHF-signals in the range of 150.000 to 150.300 MHz from 8 to 40 ms to match the specifications of the used VHF-transmitters. The detected signals were forwarded to a server system in real time and collected locally for later analyses.

Figure 2 right shows the components of the trackIT ARTS. Each station is connected to a server system running *EcoHub*, a metadata database that holds information on the locations of the stations, the orientation of their antennas, the used transmitters, tagged individuals, and ground-truth data, for example from test tracks. Whenever detection data are forwarded to the server, the respective transmitter and individual is identified using the signal information (timestamp, frequency, duration, signal strength per antenna) and written in an *InfluxDB* time series database. Detection data (raw and processed) can be viewed in real time using a set of dashboards available in the *Grafana* visualization tool. More information on hardware and software can be found in [3] and [13].

### Ground-truth data

To validate the estimated positions and derive a position error (distance between the estimated and true positions), we used ground-truth data from test tracks. For these tracks, we walked with varying pace carrying active VHF-transmitters from Plecotus Solutions GmbH, Müllheim, Germany (60 bpm, 600 *μ*W emitting power, 20 ms signal duration, 150.014–150.298 MHz frequency) fixed on a rod at different heights (0.5, 1, 1.5, 2 m above ground). The antennas of the transmitters pointed downward with approximately $45^{\circ}$ to mimic a sitting bird. We simultaneously recorded the tracks with a GPS device, optimally recording one location per second (smartphone and app GPS Logger [24]), and then matched the GPS locations to the 2-second intervals used for position estimation.

**Fig. 3:**
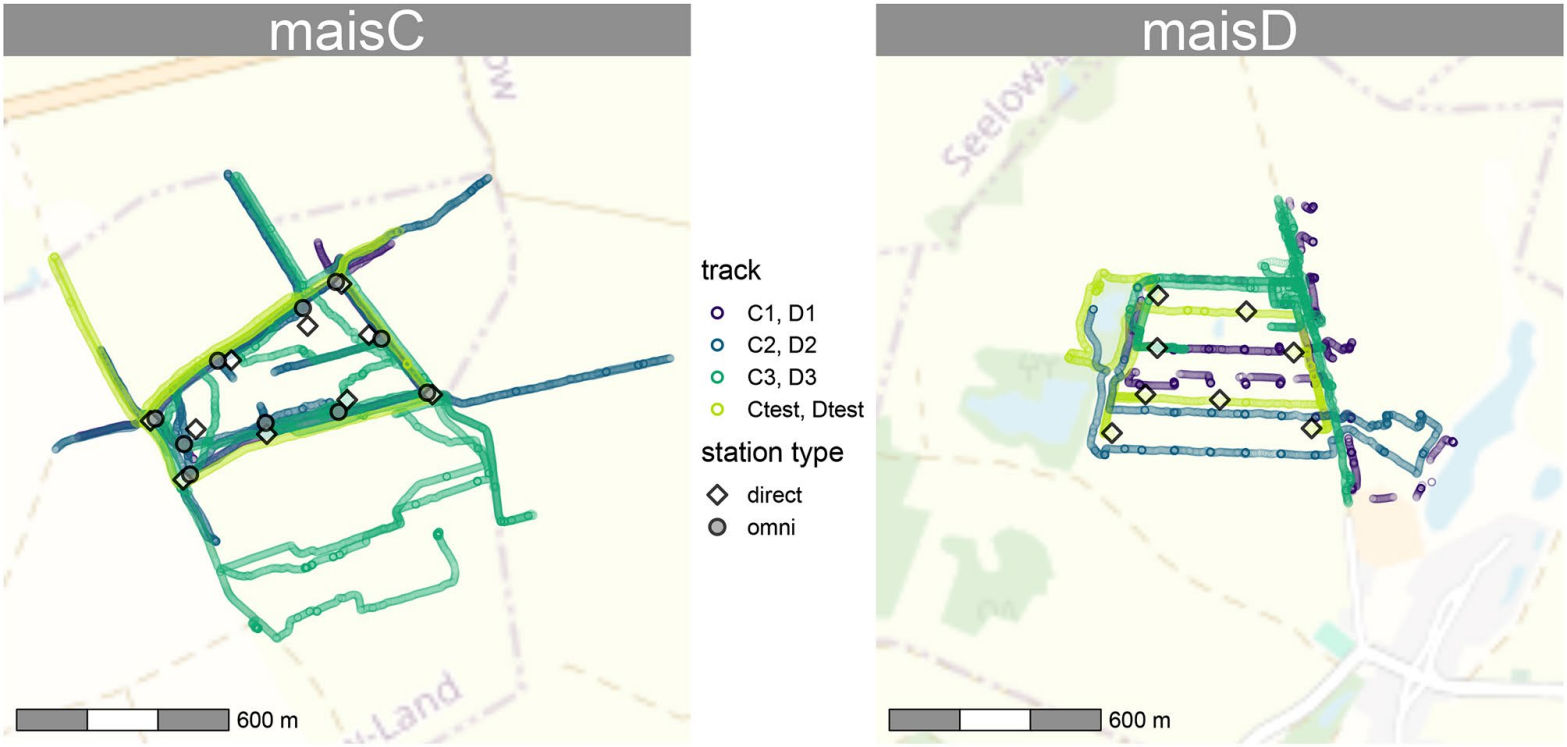
Test tracks used as ground-truth data to validate position accuracy for maisC and maisD. For properties of test tracks see Supplement 1.2. Due to issues with continuous recording, there are gaps in D1. Copyright map data: OpenStreetMap contributors

For each site, we selected four tracks for which we could ensure that all stations were running, resulting in approximately 13,500 GPS fixes per site (Fig. 3).

### Raw data filtering

**Fig. 4:**
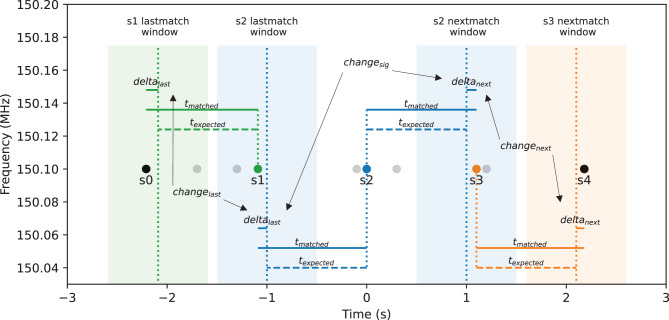
Example of *Lastmatch-Nextmatch* calculations for signals *s1, s2* and *s3*. $t_{expected}$ = expected transmitter specific time interval, $t_{matched}$ = matched signal time interval. Lightgrey points are signals (most likely false detections) that were not considered as neighboring signals because they were either positioned outside the respective time window, or another signal was closer to $t_{expected}$

To discard false positive detections, for example, due to noise from nearby power lines, we applied several filters to the recorded VHF-signals prior to position estimation. First, we used known transmitter specifications like a narrow frequency band of 4 kHz around the center frequency of each transmitter and a signal duration of 8 to 24 ms, to filter the majority of false positive detections exceeding these specifications. Second, we applied a filter based on transmitter-specific time intervals $$t_{expected}$$ between consecutive signals (here: 1 s), called *Lastmatch-Nextmatch*. The *Nextmatch* filter identified (likely) false positives by (i) calculating the deviation ($$delta_{next}$$) between the expected interval $$t_{expected}$$ and the actual interval $$t_{matched}$$ between a signal *s2* at time *t0* and its neighboring subsequent signal *s3* and (ii) calculating the deviation ($$change_{next}$$) between $$delta_{next}$$ from *s2* and $$delta_{next}$$ from *s3* (based on its interval to the subsequent signal *s4*) (Fig. 4). To be classified as a neighboring signal (*s3*), the signal must be within a given window $$((t_0 + t_{expected}) - 0.5 * t_{expected}$$, $$(t_0 + t_{expected}) + 0.5 * t_{expected})$$, if several signals meet these requirements, the signal closest to $$t_{0} + t_{expected}$$ was chosen. We implemented these steps analogously for the preceding (*Lastmatch*) signal. Finally $$change_{sig}$$ was calculated as the deviation of $$delta_{last}$$ and $$delta_{next}$$, and minimum absolute values $$(Interval\,Delta = min(abs(delta_{next}, delta_{last})$$, $$Interval\,Change = min(abs(change_{last}, change_{next}$$, $$change_{sig}))$$ were used for simple threshold-based filtering. In the context of this work, we used a threshold value of $$Interval\,Change < 0.1\,s$$. With that, all signals without at least one corresponding successor or predecessor are filtered out and practically all false detections are discarded. This filter additionally offers the advantage of adapting to the given circumstances, e.g., slightly changing transmitter-specific time intervals due to temperature, humidity, and low batteries.

### Position finding methods

To estimate positions based on automatically recorded VHF-signals, we first aggregated detected signals in 2-second intervals to account for variation in signal strength that was due to different orientation of the transmitter’s antenna (see Introduction). For position finding, we used different approaches based on (i) antenna beams (*directional antenna beams, direct ab* and *omnidirectional antenna beams, omni ab*), (ii) angulation using bearing and distance estimations (*directional angulation, direct an*), and (iii) lateration using distance estimations (*omnidirectional multilateration, omni ml*). By comparing the estimated position with the respective true position from our ground-truth data, we calculated a position error (PE). This PE was then used for optimizing and comparing the different position finding methods (Sect. 2.6.1). Moreover, by using ground-truth data we can predict PEs (pPE) based on different characteristics and transfer these predictions to estimated positions derived from transmitters without ground-truth data (e.g., a target species, Sect. 2.6.2). In this study we limit directional antenna configurations to four $90^{\circ}$ antennas. While other configurations seem useful in certain study designs this requires careful considerations when adapting the respective formulae, i.e. taking into account the antenna back lobe. For antenna beams with varying antenna configurations (more or less than four antennas and larger and smaller angles than $90^{\circ}$), see the R package movetrack developed by [22].

#### Bearing estimation

Gottwald et al. [3] described a method of bearing estimation based on perpendicularly oriented directional antennas, which we adopted as follows: For a detected signal, we selected the antenna $a_{main}$ with strongest reception $p_{main}$ and its neighboring second-strongest antenna signal $p_{second}$. The difference in gain (*Δ g*) of the antenna pair is computed and normalized using the maximum gain difference (*Δ m*) which depends on the antenna model and used transmitter:

1 $$\Delta g = \frac{p_{main} - p_{second}}{\Delta m}$$

The bearing offset ($\Delta \omega$) to the main antenna is computed as follows:

2 $$\Delta \omega = (90 - 90 * \Delta g)~/~2$$

The absolute bearing *ω* is further calculated by adding $\Delta \omega$ to the direction of the main antenna, i.e., subtracting $\Delta \omega$ in the case that $a_{second}$ is left instead of right of the main antenna:

3 $$\omega =\begin{cases}\omega_{main} + \Delta \omega, & \text{if } a_{main} < a_{second}, \\\omega_{main} - \Delta \omega, & \text{if } a_{main} > a_{second}.\end{cases}$$

#### Distance estimation

For distance estimation, we fitted an exponentially decaying curve of the form $dist = a * b^{power}$ to the actual distances calculated from a GPS-recorded calibration track (see Supplement 1.4 for an example). In the case of directional stations, we used the maximum signal strength of all four antennas, whereas for omnidirectional stations (only one antenna), we directly used the received signal strength.

#### (i) Antenna beams position finding

**Fig. 5:**
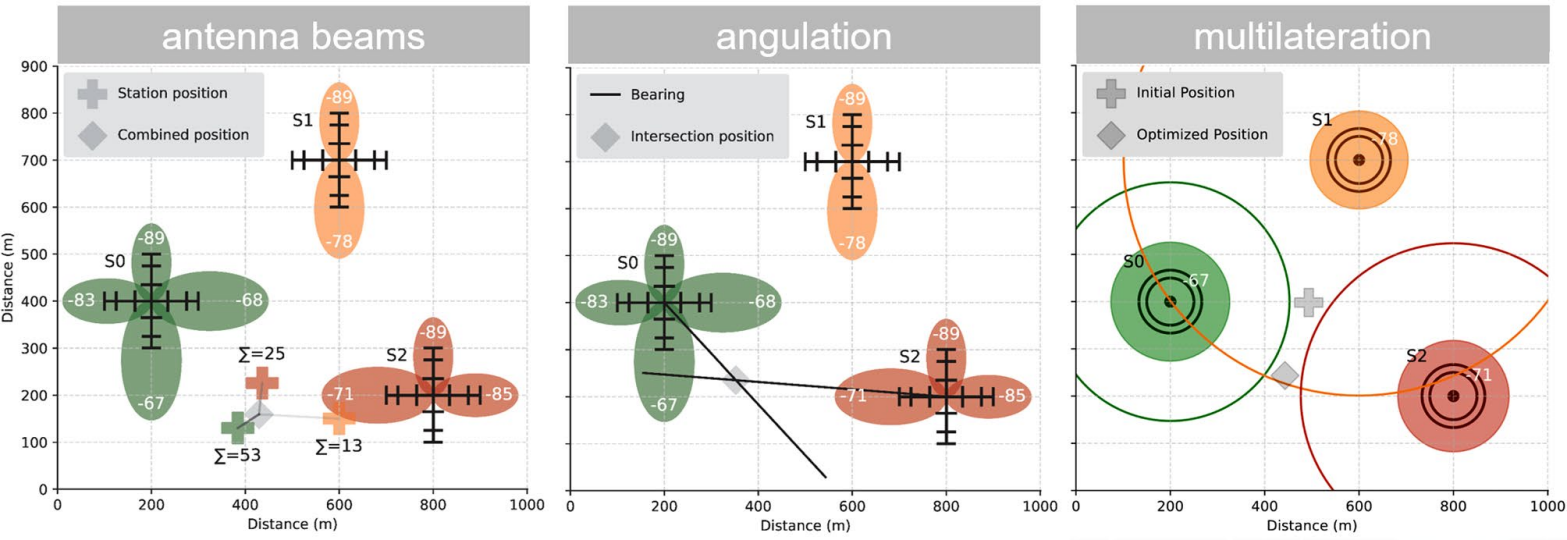
Exemplified position finding methods used in this study. Note that antenna beams were also used for omnidirectional stations. Sample calculations can be found in Supplements 1.3 to 1.7

Baldwin et al. [12] describe a geometric method for estimating coarse locations based on the expected antenna detection range of directional 9-element yagi antennas of the Motus system. This approach successfully estimates positions for large scale movements (with a consequently large position error, e.g. [22]), and is now tested for small scale movements: For each detection, a location estimate was generated along the main lobe of the receiving antenna for directional antennas, assuming a distance equal to half the detection range *r*. In the case of detection by multiple antennas within a given time interval, the resulting antenna locations were averaged using the weights of a normalized signal strength (Fig. 5, left). For omnidirectional antennas, the detection range was omitted, and we estimated positions by averaging station locations weighted by normalized signal strengths. In our study, the time interval for averaging locations was set to 2 seconds. Note that, due to the method itself, estimated positions could only fall within a defined area, namely a polygon covering all receiving stations (omnidirectional) plus a buffer of 0.5*r (directional; see Supplement 1.8).

#### (ii) Angulation position finding

Based on distance and bearing estimations, angulations using data from multiple stations were computed. Per station, we created an intersection line in the bearing direction and long as twice the least estimated distance and intersected all dual combinations of the resulting lines (Fig. 5, center). In case of several intersections, we averaged the resulting multiple angulation locations using inverse distance weighting. Restricting the length of the intersection line to twice the distance estimate prevented the estimation of unrealistic positions, i.e., intersection of lines far from the study area.

#### (iii) Multilateration position finding

Multilateration is a common method for finding a position in space based on the distance to known points. It is used, for example, in the GPS method, where the differences in transit time between signals from different satellites are used to determine position instead of distances. In this work, the distance estimates $d_s$ described in 2.5.2 were used to calculate positions for signals received with omnidirectional stations. For use with directional stations, one needs to use the strongest signal strength to estimate the distance (not included in this work). The position was estimated by first computing an initial estimate $l_0$ using the inverse distance weighted station positions $l_s$ (Fig. 5, right).

4 $$w = \sum_{s \in S} \frac{1}{d_s}$$

5 $$w_s = \frac{1}{d_s * w}$$

6 $$l_0 = \sum_{s \in S} w_s * l_s$$

Second, we optimized the position by minimizing the summed error *f(l)* of the difference in position-station distance and distance estimation:

7 $$dist(l, m) = \sqrt{(l_x - m_x)^2 + (l_y - m_y)^2}$$

8 $$f(l) = \sum_{s \in S} (dist(l - l_{s}) - d_s)^2$$

#### Station cover

Since position finding is highly influenced by where the transmitter is located and how many antennas could simultaneously receive a signal, we used a proxy for how good each position in a given study area is covered by nearby stations. We used a simple approach to calculate station cover by summing up detection probability polygons around each station. This approach assumed a linear decrease in detection probability with increasing distance to the station (−0.15 per 100 m distance), resulting in a probability of 1 within a 100 m buffer, a probability of 0.85 within a 100 m to 200 m buffer, and so forth (see Supplement 1.9). We summed up overlaying probability polygons of nearby stations, resulting in a density raster with a high station cover in the core area and a decreasing station cover towards the edges of the study site.

### Analysis

To optimize position errors (PEs), compare methods, and predict PEs for new data (pPEs), we ran generalized linear mixed models assuming a lognormal distribution (link = log) of the response variable PE, using the glmmTMB package v1.1.9 [25] in R v4.4.0 [26] and helper functions provided in [27]. Posterior predictions for pPE were done via bootstrapping by directly simulating 4000 values from the model parameters to derive i) conditional posterior predictions for specific predictor combinations and ii) observation-level posterior predictions for raw position estimates of test tracks *Ctest* and *Dtest* [27]. Per predictor combination or raw position estimate, we extracted the mean as well as the 50% and 95% confidence interval (CI) of the 4000 simulations.

#### Optimization and comparison of methods

For optimization and comparison, we ran two models:

9 *m1: PE ∼ r + (1|tagID)*

10 *m2: PE ∼ meth + (1|tagID)*

The first model (*m1*) was used to find the detection range *r* (ordered categorical, only for directional antenna beams) resulting in the lowest PE, which was then used for the second model (*m2*) to compare methods (*meth*, categorical, four in maisC, two in maisD), and to determine the method that resulted in the smallest overall PE. Both models also included transmitter ID (*tagID*, categorical) as a random intercept to account for variation between different transmitters, e.g. due to different heights or actual orientations of the transmitter’s antenna. To guarantee a balanced comparison in the second model, we used a common subset of our data reduced to timestamp and tagID combinations, where all methods were able to estimate a position.

#### Position error prediction

To predict the PE (pPE) and apply it to new data (e.g., without ground-truth data), we used ground-truth data from test tracks C1–C3 and D1–D3 to fit a model with high predictive power. Predictors were the number of participating stations (*Sc*, numeric) and antennas (*Ac*, numeric, only for directional antenna beams), the maximum received signal strength (*maxSig*, numeric), the summed up *weight* (numeric, only for antenna beams), and station *cover* (numeric). Furthermore, we used transmitter ID (*tagID*, categorical) as random intercept to account for variation between different transmitters. Values were extracted per estimated position and numerical parameters were scaled (mean = 0, SD = 1) prior to modeling. Since not all parameters were accessible for all methods, we ran model *m3.1* for directional antenna beams, *m3.2* for directional angulation and omnidirectional multilateration, and *m3.3* for omnidirectional antenna beams:

11 $$\begin{aligned}& m3.1:PE \sim Sc*Ac*cover \\ & + maxSig*weight + (1|tagID) \\ \end{aligned} $$

12 $$\begin{aligned}& m3.2:PE \sim Sc*cover \\ & + maxSig + (1|tagID) \\ \end{aligned} $$

13 $$\begin{aligned}& m3.3:PE \sim Sc*cover \\ & + maxSig*weight + (1|tagID) \\ \end{aligned} $$

The models included highly correlated parameters (Sc, Ac, cover, maxSig, weight), as well as some interactions since we were not interested in their causation, but in an optimal prediction of *PE*. We validated the predictive performance of the models by observation-level posterior predictions, i.e., predicting PEs for the two excluded tracks *Ctest* and *Dtest* via bootstrapping and comparing it to the real PEs calculating the mean absolute error (MAE). For a real life example of estimated positions and pPEs of two tagged birds, compared to positions derived from handheld telemetry see Supplement 2.5. Note that *Ac* was only included for directional antenna beams since *Ac* can be directly calculated based on *Sc* for the other methods (*Ac = Sc* for omnidirectional antenna beams and multilateration, Ac = 2*Sc for directional angulation). For directional antenna beams, *Ac* can vary between *Sc* and 4*Sc.

## Results

### Method optimization

For a site-specific optimization, we separately implemented the optimization process for maisC (four methods, directional and omnidirectional stations) and maisD (two methods, only directional stations).

#### Detection range

**Fig. 6:**
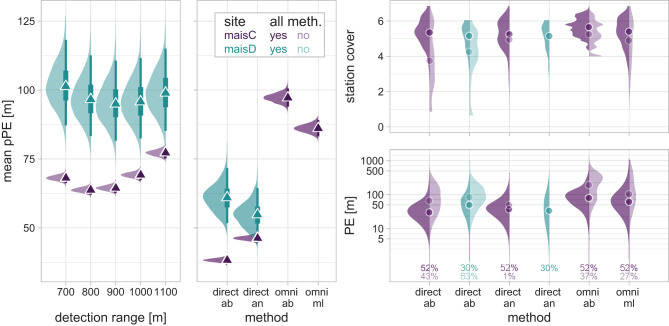
*Left, center*: Model predictions (4000 simulations, conditional posterior predictions) of method optimization and comparison. Panels show the distribution (polygons) of the mean pPE (triangle, with 50% (thick bar) and 95% (thin bar) CI) per detection range and method per site (color). *Right*: Raw data distribution (polygons) of position estimates per station cover (top) and PE (bottom). Points display median station cover and PE and widths of polygons are scaled to counts. Positions are separated based on whether they could be estimated by all methods (all meth. = “*yes*”) and were therefore used for method comparison, or not (all meth. = “*no*”). Share of estimated points to all recorded test track points is given in %. Note log10-scaling of y-axis in the bottom right panel

Concerning the detection range of directional antenna beams, the mean predicted position error (pPE) ranged between 63.8 and 77.2 m for maisC with the smallest pPE for a detection range of 800 m, while for maisD it ranged between 95.1 and 101.5 m with the smallest pPE for 900 m (Fig. 6 left). The difference in mean pPE between the detection ranges was greater and clearer in maisC than in maisD (Fig. 6 left). We continued with a detection range of 800 m (maisC) and 900 m (maisD) for method comparisons.

#### Position finding methods

When comparing methods, directional antenna beams had the lowest mean pPE (38 m) in maisC, while for maisD angulation of directional antennas (55 m) performed better than directional antenna beams (Fig. 6 center). Again, the difference in mean pPE between the methods was greater and clearer in maisC than in maisD. Note that we only included positions with estimates for all methods, which is why positions with a low station cover and therefore usually high PE were excluded more frequently, resulting in a comparable lower mean pPE for directional antenna beams when comparing methods than when comparing detection ranges (Fig. 6). Positions that were estimated by all methods were usually positioned inside the station set-up namely the core area (see also Supplement 2.1 for further details per test track).

Concerning the yield (i.e., the proportion of positions that could be estimated), positions estimated by directional angulation were usually also estimated by other methods, whereas other methods resulted in way more additional positions (Fig. 6, right, Supplement 2.1). In total, approximately 50 (maisC) and 30% (maisD) of the recorded ground-truth positions could be estimated using directional angulation, while directional antenna beams resulted in more than 90% of the recorded positions (Fig. 6, bottom right). For omnidirectional stations, antenna beams resulted in position estimates for almost 90% and for multilateration in almost 80% of the recorded positions. Note that, due to the calculation itself, the estimated positions using omnidirectional antenna beams all fall within the core area (i.e. estimates of positions outside the core area nevertheless fall within the core area), resulting in high station covers only (Fig. 6, top right, Supplement 2.1).

### Position error prediction

#### Predictive performance

**Fig. 7:**
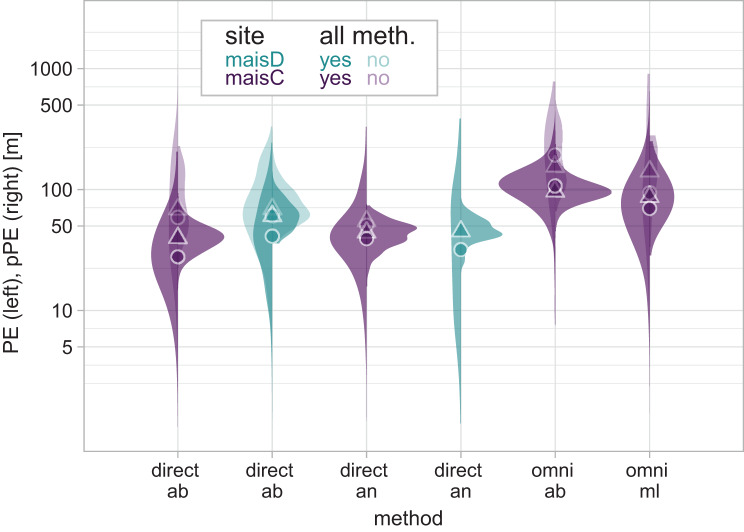
Predictive performance testing per method to predict PEs (4000 simulations, observation-level posterior predictions) for all positions from test tracks *Ctest* and *Dtest*, namely distribution of raw (left polygons, PE) and predicted PEs (right polygons, pPE) including medians (PE = points, pPE = triangles). Transparency indicates whether positions were estimated by all methods (all meth. = “*yes*”) and can be used to directly compare different methods, or not (all meth. = “*no*”) and widths of polygons are scaled to counts. Note log10-scaling of y-axis

Concerning the predictive performance of the models, i.e., the mean absolute error (MAE) between PE and pPE, the model for directional antenna beams made better or similar predictions (small MAEs: 21 m in maisC and 33 m in maisD) than for directional angulation (22 and 44 m), followed by omnidirectional antenna beams (38 m) and mutlitaleration (53 m, Table 1 case ‘all meth.’). On average, models for directional antenna beams and omnidirectional mutlilateration made more conservative predictions with pPEs being larger than real PEs, whereas pPEs from directional angulation and omnidirectional antenna beams were more optimistic and smaller than real PEs (see Table 1 case ‘all meth.’). Note that pPE showed less variation compared to PE, with only a few predictions below 20 m or above 200 m (Fig. 7).

**Table 1:** Results of predictive performance testing per method to predict PEs (4000 simulations, observation-level posterior predictions) for all positions from *Ctest* and *Dtest*, including mean *PE* (in m, raw), mean *pPE* (in m, predicted), mean absolute error *MAE* (in m), and proportion of ground-truth positions in % (*pP*) that could be estimated by the respective method. There are three different cases: *full* = all estimated positions, *all meth.* = only estimated positions present in all methods, *filtered* = only estimated positions after applying method-specific filters for Ac-Sc (see Sect. 3.2.2, for *omni ab* and *omni ml* we excluded positions with Sc * < 3*)

site	method	full				all meth.				filtered			
		PE	pPE	MAE	pP	PE	pPE	MAE	pP	PE	pPE	MAE	pP
maisC	direct ab	52	56	27	98	34	41	21	65	43	50	24	92
maisC	direct an	47	44	22	66	47	44	22	65	47	44	22	66
maisC	omni ab	145	131	48	95	110	101	38	65	122	104	40	74
maisC	omni ml	103	109	69	88	82	94	53	65	88	92	55	74
maisD	direct ab	65	72	41	99	50	63	33	39	63	69	40	97
maisD	direct an	55	47	43	39	55	47	43	39	55	47	43	39

#### Predicted PE dependencies

**Fig. 8:**
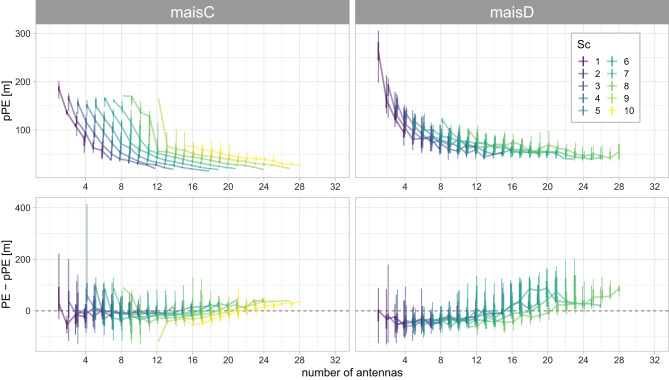
Predictive performance testing (4000 simulations, observation-level posterior predictions) using directional antenna beams based on all estimated positions from test tracks *Ctest* and *Dtest*. Values are summarized by all present Ac-Sc combinations including 95% (thin bars) and 50% CI (thick bars) and x-values are slightly shifted to prevent overlay of CIs. *Upper*: Predicted PE (pPE). *Lower*: Differences between real PE and pPE

PEs of **directional antenna beams** (for other methods and figures for conditional posterior predictions, see Supplement 2.3) varied with the covariates with deviating patterns between the two sites (Fig. 8). For simplicity, here we mainly refer to Ac and Sc but note that Ac, Sc, maxSig, cover, and weight were usually positively correlated, and therefore one has to look at the pattern in its entirety (see Supplement 2.2 for correlation plots and Supplement 2.3 for observation-level posterior prediction plots based on these predictors).

In **maisC**, the highest mean pPEs (150 to 180 m) and uncertainty occurred for combinations where *Ac = Sc* (i.e., each station received the signal with only one antenna; left end of each line in Fig. 8, top left). For the same Ac, pPE improved (= decreased) with decreasing Sc (e.g., a position estimate is more accurate if two stations each receive with three antennas than if three stations each receive with two antennas), and for the same Sc, pPE improved with increasing Ac, often approaching a pPE of 25 m or less. Concerning the predictive precision (that is the difference between raw PE and pPE) with respect to covariates, average differences were close to zero, but pPE was underestimated when more than 22 antennas were used for position estimation (Fig. 8, bottom left). Additionally, single estimates became more precise (= smaller CIs)) with increasing Ac, while there were no obvious differences between different Sc.

In **maisD**, the highest mean pPEs (150 to 270 m) and uncertainty occurred for positions recorded by few stations with few antennas (Ac-Sc combinations 1–1, 2–1, 3–1, 2–2, 3–2, 3–3, Fig. 8, top right). For the same Ac, pPE did not or only marginally improve with decreasing Sc and for the same Sc, pPE first improved with increasing Ac but then remained constant at approximately 50 m. Concerning the predictive precision with respect to covariates, pPE was overestimated for small Ac (PE * < * pPE) and underestimated for larger Ac (PE * > * pPE), (Fig. 8, bottom right). In contrast to maisC, the variance between single estimates remained more or less constant across different Ac and Sc.

Excluding position estimates with Ac-Sc combinations with high pPEs (see above) from the test tracks *Ctest* and *Dtest* resulted in a reduction of possible point estimates (6 and 2% for directional antenna beams, 21% for omnidirectional antenna beams, 14% for omnidirectional multilateration) but also in better PEs and pPEs as well as a slightly better predictive performance compared to the full dataset (Table 1, case ‘full’ vs. ‘filtered’).

## Discussion

We found substantial differences between the three position finding methods antenna beams, angulation and multilateration in terms of position errors, number of estimated positions and predictive performance. Directional stations generally produced smaller errors than omnidirectional ones, and directional antenna beams yielded substantially more estimates than angulation while having comparable position errors. Table 2 summarizes each method’s advantages and disadvantages, helping future studies to find the best method for the respective underlying study design. Per-position errors varied widely - ranging from several meters to hundreds of meters - depending on factors such as station and antenna number, station cover, signal strength, and weights. Errors were especially high outside the station set-up, underscoring the importance of predicting per-position errors rather than relying on a single average.

**Table 2:** Overview of tested methods, including pros and cons for different aspects to help selecting the best method and set-up. For a visualization of the covered area, see Supplement 1.8

	**directional** (flat, undulating)		**omnidirectional** (flat)	
	antenna beams	angulation	antenna beams	multilateration
**position error**	good (PE ${}^{\sim} 50\,\textrm{m}$ (flat), $\sim 60\,\textrm{m}$ (undulating))	good, (PE ${}^\sim 50\,\textrm{m}$)	bad (PE * > *100 m)	ok, (PE ${}^{\sim} 100\,\textrm{m}$)
**predictive performance**	good, better in flat areas, conservative estimation (pPE * > * PE)	ok, better in flat areas, optimistic estimation (pPE * < * PE)	ok, optimistic estimation (pPE * < * PE)	good, conservative estimation (pPE * > * PE)
covered area	good (core area plus buffer of 0.5*r)	on paper great, in praxis mainly restricted to core area	bad, restricted to core area	good, (core area plus buffer of maximal distance estimation)
yield	great, without filter all signals can be used	bad, at least two stations with two neighbouring antennas needed (plus intersecting bearing lines)	great, without filter all signals can be used	good, at least two stations needed
costs	more expensive		cheaper	
set-up	elaborate set-up and maintainance		simple set-up and less maintainance	

### Method optimization

#### Detection range

Concerning the best detection range to be used for directional antenna beam position estimates, the results for maisC were more pronounced, with 800 m clearly deviating from other ranges, while there was a large overlap between ranges for maisD with 900 m resulting in the smallest pPE (Fig. 6, left).

Part of the differences between the two sites might be due to the set-up and test tracks used, since they were not identical, but we expect that these results were mainly linked to the respective topography (Fig. 1). All position finding methods used in this study assumed the same detection probability and received signal strength regardless of whether the transmitter is positioned at the same distance from the receiving station in northern, eastern, southern, or western direction. Signal detection is highly dependent on signal transmission, which can vary due to the position of the transmitter antenna, whether the signal is weakened by surrounding vegetation, the level of humidity, or how fast the transmitter is moving [1, 4, 13], but this variation usually occurs randomly in any direction. Topography affects signal detection based on slope direction, with downhill-facing antennas typically achieving greater detection ranges than uphill-facing ones. Elevation differences between stations further increase variability in signal detection. Therefore, position errors in undulating landscapes vary highly within one assumed detection range, while errors between ranges remain more consistent. Our position finding method antenna beams did not account for such ‘static’ differences in signal detection due to topography, resulting in less accurate position estimates at undulating sites. Thus, more research is needed, for example, by using different detection ranges per station (and/or antenna) or applying received signal strength (RSS) fingerprinting, a machine learning approach matching received signals from unknown positions to signal fingerprints from known ground-truth positions. The latter has at least been shown to work well for omnidirectional and directional set-ups: [16] achieved a median position error of 30 m for positions between 0 and 75 m from the nearest station in a fairly dense omnidirectional set-up (100 m spacing between stations) and [14] achieved a median position error of 230 m for positions between 0 and approximately 1000 m from the nearest station in a more sparse directional set-up (500 m spacing).

#### Position finding method

The four tested methods differed in their position error and yield, i.e., the proportion of positions that could be estimated. In terms of yield, antenna beams proved to be better than angulation for directional stations with comparable pPEs, while for omnidirectional stations, multilateration resulted in smaller pPEs and a comparable yield than antenna beams (Fig. 6, center, right). The reduced yield in the angulation method arose from various prerequisites that must be met: to calculate bearings, at least two stations need to detect the signal, each with two neighboring antennas, and the resulting lines need to intersect (see Sect. 2.5.3). Consequently, a substantial number of positions could not be estimated, resulting in reduced temporal resolution. Position estimation was particularly limited when transmitters were located outside the core area, leading to a total loss of 34 to 61% of positions (Table 1, Supplement 2.1). Antenna beams, on the other hand, could even estimate positions from single detections - though with high error - (only 1 to 5% loss) while multilateration required at least two stations (12% loss). Antenna beams and multilateration thereby covered a larger area than angulation, offering a more comprehensive view of movement patterns (Supplement 2.1).

Regarding the differences between station types, there was a clear trade-off between smaller PEs (directional stations) and a more affordable and simpler station set-up (omnidirectional stations). With a good signal basis (Sc *\ge* 3 receiving with 2*Sc directional or Sc omnidirectional antennas), the two directional methods could achieve mean pPEs between 15 and 50 m, while the omnidirectional system in maisC had mean errors between 50 and 150 m for multilateration and around 100 m for antenna beams (see Fig. 8 and Supplement 2.3). Compared to previous studies using directional and/or omnidirectional set-ups, our results ranged in the midfield of measured errors (mean spacing between stations 155 to 175 m): In omnidirectional set-ups using multilateration [15] obtained mean PEs of 7 m (spacing 12 m), [16] median PEs of 43 m (spacing 100 m), [17] mean PEs of 63 m (spacing 100 m), and [20] mean PEs of 180 m (62 to 141 m after applying several filters, spacing 215 m). In directional set-ups using angulation, [3] obtained mean PEs of 25 m (spacing 200 m), [9] measured median PEs of 72 m for moving butterflys (spacing 250 m), and [14] got mean PEs of 550 m (spacing 500 m). See also Supplement 2.4 for an overview table of PEs from different studies and study designs, including results from our study. However, direct comparison between different set-ups is always difficult since errors depend on various factors such as emitting power of transmitters, where in relation to the stations the ground-truth data was recorded, spacing between stations, which and how many positions could be estimated, height above ground of antennas and transmitters, surrounding vegetation, and topography [1, 4, 9, 13].

Omnidirectional antennas usually have a smaller detection range than directional antennas. If a signal is detected by fewer stations compared to directional stations, the resulting position estimations will thus be less precise. One way to compensate for these deficiencies and improve position estimations is by decreasing the minimum distance between stations, as shown by [20]. However, this would come along with either a decrease in covered area when using the same number of stations, or the need for more stations to cover the same area, and therefore an increase in costs. An alternative would be to increase either the antenna height or the transmitting power of the radio transmitter.

### Position error prediction

#### Predictive performance

Models used to predict position errors (pPE) performed well, with mean absolute errors (MAE) between real PE and pPE for test tracks *Ctest* and *Dtest* ranging between 21 (directional antenna beams) and 69 m (omnidirectional multilateration, Table 1). Since predictions were mean estimates for given combinations of covariates, they usually overestimated extremely low PEs and underestimated high PEs. However, these extreme values occurred only rarely, which is why predictions can, on the whole, provide a reliable result.

#### Predicted PE dependencies

Positions estimated with one method varied extremely in their position errors, and this was strongly linked to covariates related to how good a signal was detected (e.g., number of receiving stations and antennas, signal strength, station cover, …). Using this information to predict a position error for each position can therefore be a powerful tool to improve results based on telemetry data. It is important to note that the set of covariates used in this study is not exhaustive and can be adapted in future research. Investigators may choose to add new covariates that are relevant to their specific study systems or exclude those that are unavailable or not informative. Furthermore, excluding positions based on thresholds of these covariates effectively minimized PEs (i.e., excluding positions with low Sc (and Ac), Table 1). For omnidirecitonal and directional antenna beams, we especially recommend excluding positions based on one antenna and station only, since these estimates were (i) extraordinarily high (especially in maisD), and (ii) showed a high uncertainty when comparing real PEs and pPEs (Fig. 8, Supplement 2.3). However, such thresholds came along with a reduction in yield, thus one has to face a trade-off between many positions and small PEs and may still be of interest depending on the target research question.

Paxton et al. [20] demonstrated that increasing the number of stations used for position estimation can degrade accuracy, causing estimates to shift toward the center of the study area, with the effect being most pronounced at the periphery. Similarly, our position estimates based on antenna beams showed a centralizing bias, and position errors were underestimated when many antennas received a signal (Fig. 8). Thus, accuracy and spatial resolution may be improved by implementing additional filtering techniques, such as excluding stations with weak signal strength, as proposed by [20]. However, a key advantage of our approach is that, despite potential inaccuracies in position estimates, the predicted position error reliably reflects the associated uncertainty and can thus be used as a proxy of the trustworthiness of the estimate.

### Transferability and recommendations

The optimal detection range, predictive performance of the per-position error model, and the influence of individual predictors on position error varied between the two study sites. This demonstrates that the error model is not directly transferable to other settings without adaptation. While it may be possible to develop a generalized error model under highly standardized conditions (e.g., identical ARTS hard- and software, station spacing, antenna configuration, and comparable environmental conditions), the effectiveness of such a model remains untested and warrants further investigation.

Therefore, we strongly recommend collecting site-specific ground-truth data to calibrate the error model for each new study. At the minimum, this should include one extensive calibration walk to train the model and one shorter validation walk to assess its performance. These walks should cover both the central area of the study site and its periphery (extending to at least the stations plus half the expected detection range). To capture the site’s heterogeneity, the spatial resolution of the track should be adapted to the landscape: finer sampling may be necessary in areas with complex or varied habitats, while more uniform landscapes may require less dense tracks. Additionally, conducting these walks with multiple tags simultaneously can further improve the robustness of the calibration. This data can then be used to find the best position finding method, estimate positions and predict position errors with the error model. Mapping these results on a spatial scale (see Supplement 2.1) helps to investigate, whether changes to the stations set-up are needed, e.g., a closer spacing of stations or the addition of stations in blind spots.

## Conclusion

Our study showed that the methods tested for position finding in ARTS differed in their position error, number of yielded positions, and predictive performance. Antenna beams used for directional stations proved to be a strong alternative to the commonly used angulation, especially in terms of yield and temporal resolution. Furthermore, position errors and performance varied between the two tested study sites and were highly influenced by signal and position characteristics. When conducting radiotelemetry studies, it is therefore crucial to record ground-truth data in the field to capture this individual PE pattern of your study site and check whether (i) the resulting mean PE meets the required position accuracy of your question of interest, (ii) the predicted PEs adequately reflect measured PEs (small MAE, difference close to 0), and (iii) the yielded number of estimated positions is sufficient. The resulting estimated positions and predicted per-position errors provide a sound basis for further high-resolution analyses of wildlife movements.

## Electronic supplementary material

Below is the link to the electronic supplementary material.

Supplementary Material 1

## Data Availability

R code and data examples are available in the Zenodo repository 10.5281/zenodo.15574140 [28]. Additional supporting information can be found in the online version of the article at the publisher’s website (’Supplement.html’).

## Abbreviations

ab: Antenna beams
an: Angulation
ARTS: Automated Radiotelemetry Systems
direct: Directional station
GPS: Global Positioning System
MAE: Mean absolute error
ml: Multilateration
omni: Omnidirectional station
PE: Position error
pPE: Predicted position error
VHF: Very-high-frequency.

## Footnotes

1 trackIT OS version 2023.05.3, available online: https://github.com/trackIT-Systems/tsOS-vhf/releases/tag/tRackIT-OS-2023.05.3

## References

[1] Kays R, Crofoot MC, Jetz W, Wikelski M. Terrestrial animal tracking as an eye on life and planet. Science. 2015;348(6240). 10.1126/science.aaa2478. 10.1126/science.aaa2478 26068858

[2] Taylor PD, Crewe TL, Mackenzie SA, Lepage D, Aubry Y, Crysler Z, et al. The Motus Wildlife Tracking System: a collaborative research network to enhance the understanding of wildlife movement. Avian Conserv Ecol. 2017;12(1). 10.5751/ace-00953-120108.

[3] Gottwald J, Zeidler R, Friess N, Ludwig M, Reudenbach C, Nauss T. Introduction of an automatic and open-source radio-tracking system for small animals. Methods Ecol Evol. 2019;10(12):2163–72. 10.1111/2041-210X.13294.

[4] Kays R, Tilak S, Crofoot M, Fountain T, Obando D, Ortega A, Kuemmeth F. Tracking animal location and activity with an automated radio telemetry system in a tropical rainforest. Comput J. 2011;54(12):1931–48. 10.1093/comjnl/bxr072.

[5] Millspaugh J, Marzluff JM, editors. Radio tracking and animal populations. Academic; 2001. Available from: 10.1016/B978-0-12-497781-5.X5000-2.

[6] Joo R, Picardi S, Boone ME, Clay TA, Patrick SC, Romero-Romero VS, et al. Recent trends in movement ecology of animals and human mobility. Mov Ecol. 2022;10(1):26. 10.1186/s40462-022-00322-9. 35614458 10.1186/s40462-022-00322-9 PMC9134608

[7] Gauld J, Atkinson PW, Silva JP, Senn A, Franco AMA. Characterisation of a new lightweight LoRaWAN GPS bio-logger and deployment on griffon vultures Gyps fulvus. Anim Biotelem. 2023;11(1). 10.1186/s40317-023-00329-y.

[8] Barron DG, Brawn JD, Weatherhead PJ. Meta-analysis of transmitter effects on avian behaviour and ecology. Methods Ecol Evol. 2010;1(2):180–87. 10.1111/j.2041-210x.2010.00013.x.

[9] Fisher KE, Dixon PM, Han G, Adelman JS, Bradbury SP. Locating large insects using automated VHF radio telemetry with a multi-antennae array. Methods Ecol Evol. 2021;12(3):494–506. 10.1111/2041-210x.13529.

[10] Kissling WD, Pattemore DE, Hagen M. Challenges and prospects in the telemetry of insects. Biol Rev. 2014;89(3):511–30. 10.1111/brv.12065. 24106908 10.1111/brv.12065

[11] Rhodes EM, Burcher S, Johnson E, Contreras-Martínez S, La Puma DA, Shepard K, et al. Ultralight solar transmitter enables fine-scale movement ecology in north American hummingbird migration. bioRxiv. 2025. 10.64898/2025.12.29.696924.

[12] Baldwin JW, Leap K, Finn JT, Smetzer JR. Bayesian state-space models reveal unobserved off-shore nocturnal migration from Motus data. Ecol Modell. 2018;386:38–46. 10.1016/j.ecolmodel.2018.08.006.

[13] Höchst J, Gottwald J, Lampe P, Zobel J, Nauss T, Steinmetz R, et al. tRackit OS: open-source software for reliable VHF wildlife tracking. In: 51. Jahrestagung der Gesellschaft für Informatik, Digitale Kulturen, INFORMATIK 2021, Berlin, Germany. LNI. GI; 2021. p. 425–42.

[14] van Osta Jm JM, Dreis B, Grogan LF, Castley JG. A location fingerprinting approach for the automated radio telemetry of wildlife and comparison to alternative methods. Anim Biotelem. 2024;12(1). 10.1186/s40317-024-00379-w.

[15] Wallace G, Elden M, Boucher R, Phelps S. An automated radiotelemetry system (ARTS) for monitoring small mammals. Methods Ecol Evol. 2022;13(5):976–86. 10.1111/2041-210x.13794.

[16] Tyson C, Fragueira R, Sansano-Sansano E, Yu H, Naguib M. Fingerprint localisation for fine-scale wildlife tracking using automated radio telemetry. Methods Ecol Evol. 2024;15(11):2118–28. 10.1111/2041-210x.14410.

[17] Burcher S, Blackshire S, Gorzo J, Lanzone M, La Puma D, Mizrahi D. Improving the spatial accuracy of wildlife tracking data with automated radio telemetry systems. Anim Biotelem. 2025;13(1):37. 10.1186/s40317-025-00431-3.

[18] Beardsworth CE, Gobbens E, van Maarseveen F, Denissen B, Dekinga A, Nathan R, et al. Validating ATLAS: a regional-scale high-throughput tracking system. Methods Ecol Evol. 2022;13(9):1990–2004. 10.1111/2041-210x.13913.

[19] Menzies D, Patison KP, Fox DR, Swain DL. A scoping study to assess the precision of an automated radiolocation animal tracking system. Comput Electron Agric. 2016;124:175–83. 10.1016/j.compag.2016.04.001.

[20] Paxton KL, Baker KM, Crytser ZB, Guinto RMP, Brinck KW, Rogers HS, et al. Optimizing trilateration estimates for tracking fine-scale movement of wildlife using automated radio telemetry networks. Ecol Evol. 2022;12(2). 10.1002/ece3.8561. 10.1002/ece3.8561 PMC8831095 35169450

[21] Rueda-Uribe C, Sargent AJ, Echeverry-Galvis M, Camargo-Martínez PA, Capellini I, Lancaster LT, et al. Tracking small animals in complex landscapes: a comparison of localisation workflows for automated radio telemetry systems. Ecol Evol. 2024;14(10). 10.1002/ece3.70405. 10.1002/ece3.70405 PMC11467373 39398634

[22] Rüppel G, Karwinkel T, Brust V, Schmaljohann H. movetrack: an R package to model flight paths from radio-telemetry networks. Methods Ecol Evol. 2026;17(4):1069–81. 10.1111/2041-210x.70273.

[23] Fleming CH, Drescher-Lehman J, Noonan MJ, Akre TSB, Brown DJ, Cochrane MM, et al. A comprehensive framework for handling location error in animal tracking data. bioRxiv. 2020. 10.1101/2020.06.12.130195.

[24] BasicAirData. GPS Logger v3.2.1 [Mobile app]. Available from: https://www.basicairdata.eu/projects/android/android-gps-logger/.

[25] Brooks ME, Kristensen K, van Benthem KJ, Magnusson A, Berg CW, Nielsen A, et al. glmmTMB balances speed and flexibility among packages for zero-inflated generalized linear mixed modeling. R J. 2017;9(2):378–400. 10.32614/RJ-2017-066.

[26] R Core Team. R: a language and environment for statistical computing. Vienna, Austria. Available from: https://www.R-project.org/.

[27] Santon M, Korner-Nievergelt F, Michiels NK, Anthes N. A versatile workflow for linear modelling in R. Front Ecol Evol. 2023;11. 10.3389/fevo.2023.1065273.

[28] Rieger MR, Schmitt J, Machin P, Musculus J, Dittrich R, Gottschalk TK, et al. Data and script of ‘enabling high-resolution wildlife tracking: antenna beam-based approach including novel per-position error estimations for automated radiotelemetry systems’. Zenodo. Available from: 10.5281/zenodo.15574140. 10.1186/s40462-026-00687-1 PMC13445756 42563175

