# Supplementary material for: Enabling high-resolution wildlife tracking: antenna beam-based approach including novel per-position error estimations for automated radiotelemetry systems
Source: Mov Ecol. 2026 Aug 5;14:55. doi: 10.1186/s40462-026-00687-1 (PMC13445756; doi:10.1186/s40462-026-00687-1)
Supplement: Supplementary file 1 [file 40462_2026_687_MOESM1_ESM.html]

Supplement of ‘Enabling High-Resolution Wildlife Tracking: antenna beam-based approach including novel per-position error estimations for Automated Radiotelemetry Systems’


# Supplement of ‘Enabling High-Resolution Wildlife Tracking: antenna beam-based approach including novel per-position error estimations for Automated Radiotelemetry Systems’

#### Rieger, M. R., Schmitt, J., Machin, P., Musculus, J., Dittrich, R., Gottschalk, T. K., Gottwald, J., Lampe, P., & Höchst, J.

# 1 Methods

## 1.1 Study area

Table 1.1: Properties of the study areas maisC and maisD, including the number of directional (*N direct*) and omnidirectional (*N omni*) stations and the mean distance to the closest station (*distance*), the area enclosed by the station set-up (*core area*) as well as the number of transmitters used for testing (*N tags*) and the height above ground they were attached (*height tags*).

| site | maisC | maisD |
| --- | --- | --- |
| location | Vierlinden, Brandenburg, Germany | Döbberin, Brandenburg, Germany |
| elevation | 6-8 m asl | 47-63 m asl |
| vegetation | agriculture, tree lines, hedges, ditches with reed | agriculture, tree lines, lakes with reed and woods |
| core area | 20 ha | 16 ha |
| N direct (distance) | 10 (175 m) | 8 (170 m) |
| N omni (distance) | 10 (155 m) | 0 |
| height of station | 6 m | 8 m |
| N testtags | 4 | 3 |
| height testtags | 0.5m, 1m, 1.5m, 2m | 0.5m, 1m, 1.5m |

## 1.2 Ground-truth data

Table 1.2: Properties of test tracks used to compare position estimations and errors for maisC (*C1-3, Ctest*) and maisD (*D1-3, Ctest*). *N points* is the number of recorded GPS fixes (summarized in 2-second intervals) and *N tags* the number of used transmitters.

| track ID | date | duration (h) | length (km) | N points | N tags |
| --- | --- | --- | --- | --- | --- |
| C1 | 2023-10-05 | 0.73 | 3.33 | 1259 | 3 |
| C2 | 2023-10-09 | 2.99 | 10.59 | 5371 | 4 |
| C3 | 2023-10-10 | 3.00 | 11.74 | 5401 | 4 |
| Ctest | 2023-10-13 | 1.05 | 5.07 | 1744 | 4 |
| D1 | 2023-09-07 | 4.32 | 7.30 | 3406 | 3 |
| D2 | 2023-09-16 | 1.81 | 3.30 | 3219 | 3 |
| D3 | 2023-09-28 | 2.36 | 6.63 | 4250 | 3 |
| Dtest | 2023-09-13 | 1.47 | 3.96 | 2646 | 3 |

## 1.3 Bearing estimation

The bearings computed using the described method (x-axis) were compared to bearings computed from the positions recorded using a GPS track (y-axis), where the color indicated the error in bearing estimation. Received signals were matched to the GPS locations using the nearest time, hence GPS locations where no signal was received were not used for calibration. In the example, 10,543 signal detections with GPS location matches resulted in a median error of 4.5° and a root mean square error (RMSE) of 8.3°. While the median error indicated that the antenna might be rotated slightly by around 4.5 during the tests, the RMSE showed the variance in bearing estimates. Specifically the bearings near perpendicular to the antenna axes, i.e., 0°, 90°, 180° and 270°, had the largest errors. This was due to falsely selecting the antenna with the second strongest signal reception, which might happen due to the reception on the back lobe of the antenna.

Figure 1.1: Example of linear bearing model applied to one station in maisC. x-axis = estimated bearings, y-axis = (true) bearings based on GPS locations from ground-truth data.

## 1.4 Distance estimation

Fig. 1.2 showed a calibration curve of an exponential power distance model fitted to the underlying calibration data. The received signals and GPS locations were matched based on the nearest time, as described in the previous section. The curve was optimized using non-linear least squares. In the shown example of the directional station, a calibration track of 7,371 detected GPS fixes achieved a median distance error of 32.7 m with a root mean square error of 49.7 m. For the omnidirectional station, a calibration track of 13,014 detected GPS fixes achieved a median distance error of 24.4 m with a root mean square error of 60.2 m. The smaller distance error for omnidirectional stations is due to their uniform antenna characteristic compared to the directional characteristic. The error in distance estimation was mainly caused by topography and vegetation in the test area as well as transmitter orientation.

Figure 1.2: Exponential power distance model fit for one directional (*left*) and one omnidirectional (*right*) station in maisC.

## 1.5 Angulation

For the example we used three directional stations \(s\_0\), \(s\_1\) and \(s\_2\) each receiving the same signal of a transmitter.
Based on the received signals strengths, \(s\_0\) selected the southern as the main and eastern as second antenna.
Using the received signal strengths and the configured maximum gain difference \(\Delta m\_ = 20\), the bearing was computed as follows:

\[
\begin{align}
\Delta \omega\_{s\_0} &= (90° - 90° \* \frac{-67 -(-68)}{20})~/~2 = 42.8° \\
\omega\_{s\_0} &= 180° - 42.8° = 137.2°
\end{align}
\]

The bearing of station \(s\_2\) was computed analog resulting in \(\omega\_{s\_2} = 274.5°\).
For \(s\_1\) no bearing could be estimated, since neither the eastern nor the western antenna detected the signal and hence could not be selected as second antenna to the main southern antenna.
In terms of distance estimates in the presented illustrative example a fixed optimized curve of \(d = 4 \* 0.94^{p}\) was used (note that in a real world application, each station would have an individually fixed model).
This results in distance estimations \(d\_{s\_0} = 4 \* 0.94^{-67} = 252.6~m\) and \(d\_{s\_2} = 4 \* 0.94^{-71} = 323.6~m\).
Bearing and distance estimations are then used to compute pairwise intersections, which yielded one intersection in the presented example.

Figure 1.3: Exemplified position finding using angulation based on bearings.

## 1.6 Antenna beams

The antenna beam computations were performed using a detection range of \(r = 1,300 m\) and a gain normalization of \(p\_n = 90 dB\).
First, the gain normalization was used to compute per-antenna weights \(W\) and the summed station weight \(w\):

\[
\begin{align}
W\_{s\_0} &= (-89, -68, -67, -83) + 90 = (1, 22, 23, 7) \\
w\_{s\_0} &= \sum W\_{s\_0} = 53
\end{align}
\]

Second, the relative positions \(\Delta x\) and \(\Delta y\) were computed using the weight difference on the respective axis divided by the station weight and multiplied with half the detection range.

\[
\begin{align}
\Delta x\_{s\_0} &= \frac{W\_{s\_0, 1} - W\_{s\_0, 3}}{w\_{s\_0}} \* \frac{p}{2} = \frac{22-7}{53} \* 650.0 = 184.0 m \\
\Delta y\_{s\_0} &= \frac{W\_{s\_0, 0} - W\_{s\_0, 2}}{w\_{s\_0}} \* \frac{p}{2} = \frac{1-23}{53} \* 650.0 = -269.8 m
\end{align}
\]

Third, the absolute locations were computed by adding the station coordinates, taking into account any configured rotation of the station.

For every station with at least one received signal, a station location and a weight value are obtained, indicated by the colored plus symbol.
Finally, using the weight \(w\) of the stations, a combined position (weighted mean) was computed illustrated by the diamond marker.

Figure 1.4: Exemplified position finding using antenna beams based on antenna geometry.

## 1.7 Multilateration

For multilateration, the distance estimates per station were computed using the aforementioned exponentially fitted function \(dist = 4 \* 0.94^{power}\).
This resulted in the distance estimates  
\(d\_{S0} = 4 \* 0.94^{-67} = 252.6 m\),  
\(d\_{S1} = 4 \* 0.94^{-78} = 499.0 m\) and  
\(d\_{S2} = 4 \* 0.94^{-71} = 323.6 m\).  
The figure shows the distances as radius for the circles drawn around the center of the stations.
An initial position is computed by the inverse distance weighted average of of the station positions.
\(x\_0 = 0.437 \* (200, 400) + 0.221 \* (600, 700) + 0.341 \* (800, 200) = (493.4, 398.1)\)
Starting at this initial position the location is optimized by minimizing the summed distance error using the Nelder-Mead method, resulting in the final position \((442.9, 243.5)\).

Figure 1.5: Exemplified position finding using multilateration based on distances.

## 1.8 Covered area

Figure 1.6: Theoretical areas where an estimated position can be located (based on the respective calculation per method). If the signal of a transmitter outside the area is detected, the estimated position will fall within the area. For directional antenna beams (direct ab) we used a detection range of 800 m for maisC and 900 m for maisD (see results section), for directional angulation (direct an) we assumed a maximum distance of 2\*450 m which matches a maximum signal strength of approx. -80 dB, and for omnidirectional multilateration (omni ml) we assumed a maximum distance of 450 m. Note that the threoretical area does not imply that all true positions within this area can be estimated by the respective method. Copyright map data: OpenStreetMap contributors

## 1.9 Station cover

Figure 1.7: Exemplified calculation of detection probability (left) and station cover (summed up detection probabilities across all stations, right) for directional antennas in maisC. Copyright map data: OpenStreetMap contributors

# 2 Results

## 2.1 Estimated positions

### maisC 0.5m

Figure 2.1: Position errors (PE) of estimated positions per testtrack (columns) and method (rows) for a test transmitter at 0.5 m above ground in maisC. Ground-truth positions are displayed and colored by PE. Positions that could not be estimated are colored in gray. Note the log10 scaling of the PE color scale.

### maisC 1m

Figure 2.2: Position errors (PE) of estimated positions per testtrack (columns) and method (rows) for a test transmitter at 1 m above ground in maisC. Ground-truth positions are displayed and colored by PE. Positions that could not be estimated are colored in gray. Note the log10 scaling of the PE color scale.

### maisC 1.5m

Figure 2.3: Position errors (PE) of estimated positions per testtrack (columns) and method (rows) for a test transmitter at 1.5 m above ground in maisC. Ground-truth positions are displayed and colored by PE. Positions that could not be estimated are colored in gray. Note the log10 scaling of the PE color scale.

### maisC 2m

Figure 2.4: Position errors (PE) of estimated positions per testtrack (columns) and method (rows) for a test transmitter at 2 m above ground in maisC. Ground-truth positions are displayed and colored by PE. Positions that could not be estimated are colored in gray. Note the log10 scaling of the PE color scale.

### maisC all meth.

Figure 2.5: Overview whether positions in maisC could be estimated by all methods (‘yes’, orange) or not (‘no’, grey) per testtrack (columns) and test transmitter (rows, the label depicts the transmitter’s height above ground in m). Note that transmitter 2 was not used for test track C1.

### maisD 0.5m

Figure 2.6: Position errors (PE) of estimated positions per testtrack (columns) and method (rows) for a test transmitter at 0.5 m above ground in maisD. Ground-truth positions are displayed and colored by PE. Positions that could not be estimated are colored in gray. Note the log10 scaling of the PE color scale.

### maisD 1m

Figure 2.7: Position errors (PE) of estimated positions per testtrack (columns) and method (rows) for a test transmitter at 1 m above ground in maisD. Ground-truth positions are displayed and colored by PE. Positions that could not be estimated are colored in gray. Note the log10 scaling of the PE color scale.

### maisD 1.5m

Figure 2.8: Position errors (PE) of estimated positions per testtrack (columns) and method (rows) for a test transmitter at 1.5 m above ground in maisD. Ground-truth positions are displayed and colored by PE. Positions that could not be estimated are colored in gray. Note the log10 scaling of the PE color scale.

### maisD all meth.

Figure 2.9: Overview whether positions in mais D could be estimated by all methods (‘yes’, orange) or not (‘no’, grey) per testtrack (columns) and test transmitter (rows, the label depicts the transmitter’s height above ground in m).

## 2.2 Correlations between predictors

### direct ab - maisC

Figure 2.10: Correlations between predictors (Ac = number of receiving antennas, Sc = number of receiving stations, cover = station cover (proxy for detection probability), maxSig = maximum received signal (dB), Weight = summed up normalized signals), including correlation coefficients (upper panels) and histograms (diagonal panels) for directional antenna beams in maisC. For comparison, the log10-scaled response variable PE is given as well (PE\_log10).

### direct ab - maisD

Figure 2.11: Correlations between predictors (Ac = number of receiving antennas, Sc = number of receiving stations, cover = station cover (proxy for detection probability), maxSig = maximum received signal (dB), Weight = summed up normalized signals), including correlation coefficients (upper panels) and histograms (diagonal panels) for directional antenna beams in maisD. For comparison, the log10-scaled response variable PE is given as well (PE\_log10).

### direct an - maisC

Figure 2.12: Correlations between predictors (Sc = number of receiving stations, cover = station cover (proxy for detection probability), maxSig = maximum received signal (dB)), including correlation coefficients (upper panels) and histograms (diagonal panels) for directional angulation in maisC. For comparison, the log10-scaled response variable PE is given as well (PE\_log10).

### direct an - maisD

Figure 2.13: Correlations between predictors (Sc = number of receiving stations, cover = station cover (proxy for detection probability), maxSig = maximum received signal (dB)), including correlation coefficients (upper panels) and histograms (diagonal panels) for directional angulation in maisD. For comparison, the log10-scaled response variable PE is given as well (PE\_log10).

### omni ab - maisC

Figure 2.14: Correlations between predictors (Sc = number of receiving stations, cover = station cover (proxy for detection probability), maxSig = maximum received signal (dB), Weight = summed up normalized signals), including correlation coefficients (upper panels) and histograms (diagonal panels) for omnidirectional antenna beams in maisC. For comparison, the log10-scaled response variable PE is given as well (PE\_log10).

### omni ml - maisC

Figure 2.15: Correlations between predictors (Sc = number of receiving stations, cover = station cover (proxy for detection probability), maxSig = maximum received signal (dB)), including correlation coefficients (upper panels) and histograms (diagonal panels) for omnidirectional multilateration in maisC. For comparison, the log10-scaled response variable PE is given as well (PE\_log10).

## 2.3 Position error prediction

### pPE model

Figure 2.16: Predicted mean PE (pPE, based on 4000 simulations) from the model, namely the median pPE (triangle) including 50 % (thick bar) and 95 % CI (thin bar), as well as the distribution (polygon, only center and bottom panels) for all present combinations of Ac-Sc per site and method. For conditional posterior predictions, maxSig, (weight) and cover were set to their respective raw data mean per Ac-Sc combination.

### pPE Ctest, Dtest

#### Ac

Figure 2.17: Predicted PE (pPE, based on 4000 simulations) for position estimates from test tracks *Ctest* and *Dtest*, namely the median pPE (triangle) including 50 % (thick bar) and 95 % CI (thin bar), as well as the distribution (polygon, only center and bottom panels). Positiones are summarized per present combinations of Ac-Sc per site and method. Note that this is based on raw data (observation-level posterior predictions), therefore showing more variance and larger CIs.

#### maxSig

Figure 2.18: Predicted PE (pPE, based on 4000 simulations) for position estimates from test tracks *Ctest* and *Dtest*, namely the median pPE (triangle) including 50 % (thick bar) and 95 % CI (thin bar). Positiones are summarized per present combinations of maximum signal strength (rounded to tens) and Sc per site and method. Note that this is based on raw data (observation-level posterior predictions), therefore showing more variance and larger CIs.

#### weight

Figure 2.19: Predicted PE (pPE, based on 4000 simulations) for position estimates from test tracks *Ctest* and *Dtest*, namely the median pPE (triangle) including 50 % (thick bar) and 95 % CI (thin bar). Positiones are summarized per present combinations of summed up normalized signals (rounded to fifties) and Sc per site and method antenna beams. Note that this is based on raw data (observation-level posterior predictions), therefore showing more variance and larger CIs.

#### cover

Figure 2.20: Predicted PE (pPE, based on 4000 simulations) for position estimates from test tracks *Ctest* and *Dtest*, namely the median pPE (triangle) including 50 % (thick bar) and 95 % CI (thin bar). Positiones are summarized per present combinations of station cover (rounded to ones) and Sc per site and method. Note that this is based on raw data (observation-level posterior predictions), therefore showing more variance and larger CIs.

### diff PE - pPE Ctest, Dtest

#### Ac

Figure 2.21: Difference between raw PE and predicted PE (pPE, based on 4000 simulations) for position estimates from test tracks *Ctest* and *Dtest*, namely the median difference (triangle) including 50 % (thick bar) and 95 % CI (thin bar), as well as the distribution (polygon, only center and bottom panels). Positiones are summarized per present combinations of Ac-Sc per site and method. Note that this is based on raw data (observation-level posterior predictions), therefore showing more variance and larger CIs.

#### maxSig

Figure 2.22: Difference between raw PE and predicted PE (pPE, based on 4000 simulations) for position estimates from test tracks *Ctest* and *Dtest*, namely the median difference (triangle) including 50 % (thick bar) and 95 % CI (thin bar). Positiones are summarized per present combinations of maximum signal strength (rounded to tens) and Sc per site and method. Note that this is based on raw data (observation-level posterior predictions), therefore showing more variance and larger CIs.

#### weight

Figure 2.23: Difference between raw PE and predicted PE (pPE, based on 4000 simulations) for position estimates from test tracks *Ctest* and *Dtest*, namely the median difference (triangle) including 50 % (thick bar) and 95 % CI (thin bar). Positiones are summarized per present combinations of summed up normalized signals (rounded to fifties) and Sc per site and method antenna beams. Note that this is based on raw data (observation-level posterior predictions), therefore showing more variance and larger CIs.

#### cover

Figure 2.24: Difference between raw PE and predicted PE (pPE, based on 4000 simulations) for position estimates from test tracks *Ctest* and *Dtest*, namely the median difference (triangle) including 50 % (thick bar) and 95 % CI (thin bar). Positiones are summarized per present combinations of station cover (rounded to ones) and Sc per site and method. Note that this is based on raw data (observation-level posterior predictions), therefore showing more variance and larger CIs.

### pPE model q50

Figure 2.25: Predicted median PE (pPE50, based on 4000 simulations) from the model, namely the median pPE (triangle) including 50 % (thick bar) and 95 % CI (thin bar), as well as the distribution (polygon, only center and bottom panels) for all present combinations of Ac-Sc per site and method. For conditional posterior predictions, maxSig, (weight) and cover were set to their respective raw data mean per Ac-Sc combination. This could be done for any desired quantile (e.g. 65 % quantile instead of 50 % quantile).

### pPE Ctest, Dtest q50

Figure 2.26: Predicted median PE (pPE50, based on 4000 simulations) for position estimates from test tracks *Ctest* and *Dtest*, namely the median pPE (triangle) including 50 % (thick bar) and 95 % CI (thin bar), as well as the distribution (polygon, only center and bottom panels). Positiones are summarized per present combinations of Ac-Sc per site and method. Note that this is based on raw data (observation-level posterior predictions), therefore showing more variance and larger CIs.

## 2.4 Position error overview

Table 2.1: Overview of position errors (PE) of recent studies using ARTS with different position estimation methods in comparison with our study. PEs are given as mean and/or median. The number of directional antennas per station is given in brackets. To set the PE in relation to the used station set-up, average distance between station (spacing), station height, type of testtag procedure (stationary at points or moving along a track), as well as the covered area (core area = within the station set-up, all = including perimeter outside the set-up) are given as well.

|  | station | | |  | | | PE | |
| --- | --- | --- | --- | --- | --- | --- | --- | --- |
| study | antenna type | spacing [km] | height [m] | testtag | area covered | method | mean [m] | median [m] |
| Rüppel et al. 2026 | directional (3-4) | 1-20 | 10.0 | moving | all | antenna beams | NA | 2757 |
| Fisher et al. 2020 | directional (4) | 0.250 | 7.0 | stationary | core area | angluation | NA | 15 |
| Fisher et al. 2020 | directional (4) | 0.250 | 7.0 | stationary (butterfly) | core area | angluation | NA | 72 |
| Gottwald et al. 2019 | directional (4) | 0.200 | 2.5 | stationary | core area | angluation | 21-25 | NA |
| van Osta et al. 2024 | directional (4) | 0.500 | 4.0 | stationary (bird) | all | RSS fingerprinting | 308 | 230 |
| van Osta et al. 2024 | directional (4) | 0.500 | 4.0 | stationary (bird) | all | linear regression | 335 | 280 |
| van Osta et al. 2024 | directional (4) | 0.500 | 4.0 | stationary (bird) | all | (bi)angulation | 550 | 540 |
| Kays et al. 2011 | directional (6) | 0.800 | 40.0 | moving | all | angluation | 42 | NA |
| Kays et al. 2011 | directional (6) | 0.800 | 40.0 | moving | core area | angluation | 73 | NA |
| Beardsworth et al. 2022 | omnidirectional | 3.000 | NA | stationary | core area | Time of arrival | NA | 4 |
| Beardsworth et al. 2022 | omnidirectional | 3.000 | NA | mobile | core area | Time of arrival | NA | 6 |
| Burcher et al. 2025 | omnidirectional | 0.100 | 2.5 | moving | all | grid search algorithm | 25 | NA |
| Burcher et al. 2025 | omnidirectional | 0.100 | 2.5 | moving | all | multilateration | 63 | NA |
| Menzies et al. 2016 | omnidirectional | 0.200 | NA | stationary | core area | Time of arrival | 22 | NA |
| Paxton et a. 2022 | omnidirectional | 0.215 | 9.0 | stationary | core area | multilateration, filters | 62 | 50 |
| Paxton et a. 2022 | omnidirectional | 0.215 | 9.0 | stationary | core area | multilateration | 181 | 172 |
| Ruede-Uribe et al. 2024 | omnidirectional | 0.150 | 2.5 | moving | core area | trilateration | 116 | 105 |
| Tyson et al. 2024 | omnidirectional | 0.100 | 2.0 | stationary | core area | multilateration | NA | 43 |
| Tyson et al. 2024 | omnidirectional | 0.100 | 2.0 | stationary | core area | RSS fingerprinting | NA | 30 |
| Wallace et al. 2021 | omnidirectional | 0.012 | NA | stationary | all | trilateration | 7 | 4 |
| this study | directional (4) | 0.165 | 6.0 | moving | core area | antenna beams | 34-50 | NA |
| this study | directional (4) | 0.165 | 6.0 | moving | core area | angulation | 47-55 | NA |
| this study | directional (4) | 0.165 | 6.0 | moving | all | antenna beams | 52-65 | NA |
| this study | omnidirectional | 0.170 | 8.0 | moving | core area | multilateration | 82 | NA |
| this study | omnidirectional | 0.170 | 8.0 | moving | all | multilateration | 103 | NA |
| this study | omnidirectional | 0.170 | 8.0 | moving | core area | antenna beams | 110 | NA |
| this study | omnidirectional | 0.170 | 8.0 | moving | all | antenna beams | 145 | NA |

## 2.5 Bird data example

We also used data from one Great Tit *Parus major* and one European Robin *Erithacus rubecula* that were tagged in the course of the project to test whether the methods used can also be applied to real data.
We collected the respective ground-truth data with handheld antennas and manual angulation in the field, estimating a position at least every ten minutes for one day.
As there was rarely visual contact with the bird, these positions only served as a rough estimate of where the bird was.
For the trapping, handling and tagging of birds, authorizations were issued by the State Office for Labor Protection, Consumer Protection and Health, Brandenburg (LAVG, 2347-80-2023-9-G) and the State Office for the Environment, Brandenburg (LfU).
For animal tagging we used the same transmitters as for test tracks and ensured that transmitter weights (0.37 g, 0.6 g) did not exceed 3 % of the animal’s body weight.

Visual comparison of position estimation and error prediction with data from a tagged Great Tit and European Robin revealed a close match between positions derived from handheld telemetry and positions derived from our ARTS using directional antenna beams, but positions spread further when estimated with antenna beams.
Further, pPEs were larger for positions that were farther away from the respective handheld positions.

Figure 2.27: Estimated positions using directional antenna beams (empty symbols, size is scaled to pPE) and positions located with handheld antennas (filled symbols) recorded in one day for two individuals in maisC (Great Tit, European Robin).
